# Cardiovascular Disease Screening in Primary School Children

**DOI:** 10.3390/children12010038

**Published:** 2024-12-29

**Authors:** Alena Bagkaki, Fragiskos Parthenakis, Gregory Chlouverakis, Emmanouil Galanakis, Ioannis Germanakis

**Affiliations:** School of Medicine, University of Crete, 71 003 Heraklion, Crete, Greece; medp2011936@med.uoc.gr (A.B.); fparth@med.uoc.gr (F.P.); gchlouve@uoc.gr (G.C.); emmgalan@uoc.gr (E.G.)

**Keywords:** cardiovascular disease, screening, child, primary school, 12-lead ECG, preparticipation screening

## Abstract

Background: Screening for cardiovascular disease (CVD) and its associated risk factors in childhood facilitates early detection and timely preventive interventions. However, limited data are available regarding screening tools and their diagnostic yield when applied in unselected pediatric populations. Aims: To evaluate the performance of a CVD screening program, based on history, 12-lead ECG and phonocardiography, applied in primary school children. Methods: The methods used were prospective study, with voluntary participation of third-grade primary school children in the region of Crete/Greece, over 6 years (2018–2024). Personal and family history were collected by using a standardized questionnaire and physical evaluation (including weight, height, blood pressure measurement), and cardiac auscultation (digital phonocardiography (PCG)) and 12-lead electrocardiogram (ECG) were recorded at local health stations (Phase I). Following expert verification of responses and obtained data, assisted by designated electronic health record with incorporated decision support algorithms (phase II), pediatric cardiology evaluation at the tertiary referral center followed (phase III). Results: A total of 944 children participated (boys 49.6%). A total of 790 (83.7%) had Phase I referral indication, confirmed in 311(32.9%) during Phase II evaluation. Adiposity (10.8%) and hypertension (3.2%) as risk factors for CVD were documented in 10.8% and 3.2% of the total population, respectively. During Phase III evaluations (*n* = 201), the majority (*n* = 132, 14% of total) of children were considered as having a further indication for evaluation by other pediatric subspecialties for their reported symptoms. Abnormal CVD findings were present in 69 (7.3%) of the study population, including minor/trivial structural heart disease in 23 (2.4%) and 17 (1.8%), respectively, referred due to abnormal cardiac auscultation, and ECG abnormalities in 29 (3%), of which 6 (0.6%) were considered potentially significant (including 1 case of genetically confirmed channelopathy-LQT syndrome). Conclusions: CVD screening programs in school children can be very helpful for the early detection of CVD risk factors and of their general health as well. Expert cardiac auscultation and 12-lead ECG allow for the detection of structural and arrhythmogenic heard disease, respectively. Further study is needed regarding performance of individual components, accuracy of interpretation (including computer assisted diagnosis) and cost-effectiveness, before large-scale application of CVD screening in unselected pediatric populations.

## 1. Introduction

Screening programs for cardiovascular heart disease (CVD) in childhood aim to detect the presence of previously undiagnosed structural (congenital heart disease (CHD)) or arrhythmogenic diseases potentially associated with sudden cardiac death (SCD), as well as of risk factors of late-onset adult heart disease (such as coronary artery disease and hypertension), with increasing morbidity and mortality worldwide [1], due to an increasing impact of related risk factors already from a younger age [2]. Early detection of adult CVD risk factors offers the potential of early lifestyle modifications [3]. Identifying potential factors for the development of CVD during screening in children can potentially reduce the risk of developing a pathology that is the leading cause of mortality in modern society and the state costs allocated for its treatment and secondary prevention. Most screening programs in childhood and adolescence focus on the detection of CVD associated with SCD in specific pediatric populations such as athletes, due to an increased health risk if undiagnosed [4,5,6]. Large-scale CVD screening programs in unselected pediatric populations, although offering a potential benefit for all children [7,8], require further evaluation due to the rarity of inherited and arrhythmogenic HD in the pediatric population [9], before their large-scale application.

The present study reports the results of the only CVD screening program in Greece in unselected pediatric populations (primary school children) evaluated by the use of personal and family history questionnaires, clinical evaluation (including digital phonocardiography) and a 12-lead ECG recording. Our study findings are presented within the context of a detailed literature review regarding CVD screening in children.

## 2. Materials and Methods

This was a prospective study, with voluntary participation of third-grade primary school children (8–9 years old) in the region of Crete, Greece, over 6 years (2018–2024). The study protocol has been approved by 7th Health District Authority (Region of Crete), Hellenic Ministry of Health (532/2.8.2017 and 38890/20.9.2021) and complies with Declaration of Helsinki guidelines. Informed written consent by parents was obtained for the evaluation of participant children at regional public health facilities, after their daily school program.

### 2.1. Study Participants

A random sample of 150 schools (48% of total) was selected, representing both rural (*n* = 102) and urban (*n* = 38) regional areas of the island, with a total number of 2500 third-grade school children being eligible for participation. Parents were provided, in advance, informative material regarding the aims of the study with the support of local school authorities. The evaluation of children was performed following informed written consent provided by their parents at regional public health facilities, following completion of their daily school program.

### 2.2. Research Group

The research group performing the primary CVD screening at the local health facilities included a pediatrician with expertise in pediatric cardiology (primary investigator A.B), assisted by medical personnel or nurses having completed certified training in pediatric cardiac auscultation and pediatric ECG interpretation [10,11]. The final diagnostic evaluation, for children having referral indication following primary CVD screening, was performed in the local tertiary referral center (Pediatric Cardiology Unit, Dpt of Pediatrics, University Hospital of Heraklion) by a single academic pediatric cardiologist (I.G).

### 2.3. CVD Screening Implementation

#### 2.3.1. Phase I: Primary CVD Screening

A comprehensive CVD screening was performed, including history, physical evaluation and 12-lead ECG recording as follows.

A. Personal and family CVD history questionnaire

A specifically designed questionnaire sheet, based on preparticipation screening guidelines [4,12], was completed by parents. Personal history included presence of symptoms such as exercise intolerance, thoracic pain and perception of palpitations, history of syncope, or presence of further health issues. Family history included sudden death in family members at a young age (<50 years), and congenital or inherited CVD (cardiomyopathy/channelopathy). Positive responses were clarified through parental interviews conducted by the primary investigator during the child’s initial evaluation.

B. Physical evaluation

*Body Size.* Weight (kg) and height (cm) measurements were performed, with body mass index (BMI) estimation (BMI = kg/m^2^) and BMI percentile classification [13]. Obesity was documented as BMI ≥ 95th percentile. *Blood Pressure.* Systolic (SBP) and diastolic (DBP) blood pressure was measured using an oscillometric sphygmomanometer (Dinamap Pro Care 400 GE Medical Systems, GE Medical Systems Ltd.(Dinamap), Amersham Place, Little Chalfont, Buckinghamshire HP79NA, UK) and appropriately sized cuff. BP category according to the sex, age, and height was documented [14]. Children with BP values ≥ 90th percentile were re-evaluated repeatedly on the same day to verify measurements; femoral pulses were evaluated in all children. Stage 1 hypertension was documented in cases of average SBP and/or DBP > 95th percentile [14]. *Phenotype*: The presence of clinical features suggestive of genetic conditions potentially associated with increased CVD risk (Marfan/Noonan syndrome, hyperelasticity syndrome) were documented [12]. *Cardiac auscultation*: Conventional cardiac auscultation including dynamic auscultation (in lying and standing position) was performed for documenting the presence of a murmur (classified as innocent or abnormal) or click, detection of heart tone abnormalities, or arrhythmia (other than respiratory) [12]. *Digital Phonocardiography.* A series of 5 digital phonocardiogram recordings corresponding to the 4 standard cardiac auscultation positions and fossa jugularis were recorded in each patient, by using sensor-based electronic stethoscope with incorporated synchronous 3-channel ECG recording (The Stethoscope^®^; Welch Allyn Elite Electronic Stethoscope, Welch Allyn Inc, 4341 State Street Road, PO Box 220, Skaneateles Falls, NY 13153-0220, USA) as previously described [15]. A designated software (Meditron Analyzer 4^®^;The Meditron player 4.0. software by Meditron AS., Georgetown, Guyana) was used for data storing on a personal PC and for their offline evaluation [15].

*12-lead electrocardiogram (ECG).* A standard 12-lead ECG was recorded along with a printout of auto-measurements of intervals and individual leads’ amplitudes [16].

#### 2.3.2. Phase II. Data Verification

Cases with a negative personal and family history having an otherwise normal physical evaluation and 12-lead ECG interpretation, as evaluated by the primary investigator during Phase I evaluation, received a health clearance certificate. Phase II data validation included the confirmation of history responses, along with the expert validation of ambiguous cardiac auscultation findings and ECG recordings by a senior investigator (I.G). Digital phonocardiogram findings were evaluated offline and confirmed by an expert [15] while the use of an Electronic Health Record with computerized decision support tools (EHR- CDSS), specifically designed for our research program purposes [17], provided further assistance in the interpretation of ECG measurements along with expert final confirmation. Cases with Phase II-verified referral indication were offered an appointment in the tertiary referral center according to findings (latest within a 3-month period). Main referral indications included (a) verified positive history responses; (b) abnormal physical evaluation findings (including hypertension, adiposity, phenotypic features and abnormal auscultatory findings other than respiratory arrhythmia, respiratory S2 split and innocent heart murmur); (c) 12-lead ECG abnormalities such as left axis deviation, right axis deviation, right ventricular hypertrophy, left ventricular hypertrophy, right/left atrial enlargement, ventricular ectopic beats, pathologic Q waves, ST-T changes, T waves inversion, QTc prolongation > 460 ms, pre-excitation pattern, atrioventricular block 1st–3rd degree, complete right bundle branch block, low atrial rhythm, in accordance with prior used criteria in pediatric ECG screening programs [7,11]. A dominant referral indication was documented in cases with multiple indications, corresponding to the one considered having the highest probable association with the presence of CVD, according to investigators.

#### 2.3.3. Phase III. Tertiary Center Evaluation

A complete transthoracic echocardiographic study and a repeated 12-lead ECG were performed and evaluated by an expert pediatric cardiologist (I.G), along with physical evaluation and re-confirmation of history responses. Further imaging methods (CMR, chest radiography), 24 h ECG ambulatory monitoring, standard blood chemical analysis and genetic evaluation were performed as indicated. A further referral for invasive diagnosis or electrophysiology study was considered in selected cases. Cases whose symptoms were considered to be of non-cardiac origin were referred to further pediatric subspecialties accordingly.

### 2.4. Statistical Methods

Baseline characteristics were presented in absolute as count and relative frequencies with percentages for categorical variables. Continuous variables were reported as median, mean and interquartile range. A Chi-square test with continuity correction was used to examine the association between categorical variables. Differences in percentages were tested using Fisher’s exact test. A potential seasonal and regional variability on the incidence of CVD prevalence and risk factors was evaluated, while the diagnostic yield of phonocardiography vs. ECG screening was compared. IBM SPSS Statistics 26 software (IBMSPSS Statistics V26) was used. Reported *p* values were based on two-sided tests, and *p* values < 0.05 were considered statistically significant. CIs were calculated by a web-based calculator (https://sample-size.net/confidence-interval-proportion/, accessed on 20 October 2024).

## 3. Results

### 3.1. Baseline Characteristics

A total of 944 children (boys 49.6%, girls 50.4%), 8–9 years old (mean age 8.5 years), participated (37% of the eligible population), out of which 622 (66%) were of rural/suburban and 322 (34%) of urban residence.

### 3.2. Referral Indications

**Figure 1:**
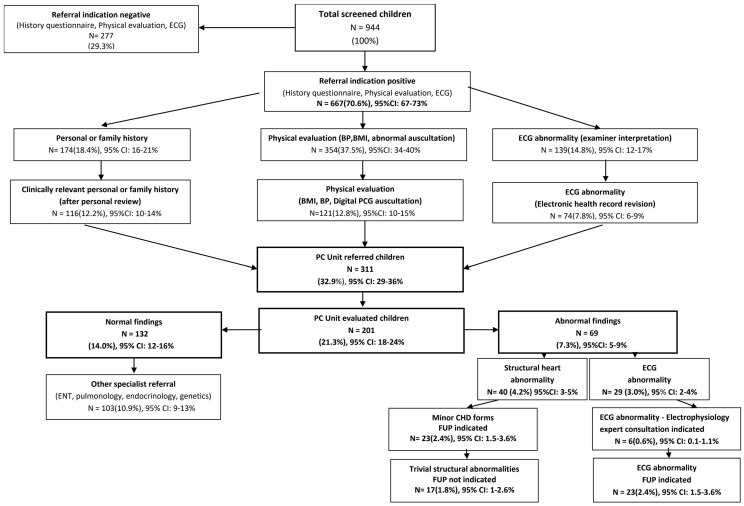
Flow diagram. CVD screening in primary school children. Referral indications and final diagnoses.

The results of the CVD screening program in children regarding referral indications and final diagnosis (Phase I–III) are summarized as a flow diagram on Figure 1.

According to the initial unconfirmed documentation (phase I), 83.7% of screened children had at least one referral indication, with 10% having multiple indications. In descending order, they included abnormal physical evaluation findings in 42.5%, (abnormal auscultation 28.2%, adiposity 10.8% and hypertension 3.2%), positive history responses in 26.4% (personal history 20.8%, family history 11.5%) and ECG abnormalities in 14.8%.

Following data validation (phase II), 311 children (32.9% of total) had a confirmed referral indication including, in descending order, abnormal physical findings 12.8%, (adiposity 6.3%, abnormal auscultation 3.9%, and hypertension 3.2%), positive history responses 12.2% (personal history 6%, family history 6.2%), and abnormal ECG 7.8%.

### 3.3. Outcomes

A final diagnostic evaluation (Phase III) was offered in 201 children (21.3% of total, 64.6% of cases with confirmed referral indication). Of them, 132 cases (14% of total) were diagnosed as normal, while in 103 (10.9%), a referral indication to other pediatric specialists was established (including ENT, pulmonology, endocrinology, genetics). A CVD diagnosis was first established in 69 children (7.3% of total, 34% of Phase II referrals), including (a) structural heart abnormalities in 40 (4.2%), all of them corresponding to minor congenital heart disease forms (bicuspid aortic valve, atrial septal defect, coronary fistula, mitral valve prolapse, mild aortic arch hypoplasia/dilation, mitral valve regurgitation) with follow-up indication in 23 cases (2.4%); and (b) ECG abnormalities in 29 (3% of total), with 6 cases (0.6% of total) having an indication for expert electrophysiology consultation and/or electrophysiology study (including pre-excitation (*n* = 1), ventricular extrasystolic arrhythmia (*n* = 4) and QTc prolongation (*n* = 1) due to a novel KCNH2 pathogenic mutation detected in the child and family members following cascade screening).

Significant temporal–spatial factors for adiposity prevalence included year of evaluation (5% vs. 9.6% before and after 2020, respectively, O.R = 2.04, *p* = 0.008) and rural residency (8.2 vs. 3.7% rural vs. urban, O.R 2.3, *p* = 0.005), while a seasonal trend for increased adiposity prevalence during autumn did not reach statistical significance. No significant temporal–spatial factors were documented for hypertension, while clusters of increased prevalence of structural heart disease detected by abnormal auscultation and echocardiography were documented in rural areas (5.4% vs. 2.7% in rural vs. urban, O.R 2.03, *p* = 0.44), while non-significant trends of both abnormal ECG and structural CVD were documented at province and school levels.

### 3.4. Diagnostic Yield of CVD Screening Components

Confirmed personal (6%) and family (6.2%) responses resulted in the diagnosis of mild, minor structural abnormalities (considered as incidental findings) in 3 (4.7%) and 7 cases (16%) of those evaluated (53–56%), respectively. While 13 (30%) of children with positive family history were considered as having some indication for long-term follow-up and/or cascade genetic screening, children with positive personal history (symptoms) were considered as being of non-cardiac origin, with 82% requiring further non-cardiology consultation (mainly pulmonology and dietology–endocrinology). Confirmed abnormal auscultation (3.9%) resulted in positive echocardiographic findings in 70% of cases including minor CHD (32%) and incidental variants (38%) of those finally evaluated (83%). The 12-lead ECG confirmed abnormalities (7.8%), including 2 cases with potential association to SCD (0.2% of the total population) among those evaluated (93%). There was no overlap of cases having both abnormal echocardiographic and 12-lead ECG findings in the present study.

### 3.5. Cost-Effectiveness Analysis 

Main cost determinants include (a) personnel (expertise, time) and (b) material costs. Phase I: Participation of trained nurses/paramedics [18]. The average evaluation time per child: 10 min (range: 8–12 min), totaling 1000 person-hours for 6,000 children. Phase II: Pediatric cardiology expertise is required; EHR documentation time: 6 min/child [17] 600 person-hours total. Phase III: Expert evaluation takes on average 20 min; 740 person-hours required for 37% referred cases. Costs: Personnel: 40,000€/year (transfer and further subsistence costs included). Materials: 10,000€/year ( hardware/software and consumables included). The implementation of paperless documentation forms (such as designated apps) or computer-based auto-interpretation of pediatric ECGs and digital phonocardiograms [19] could improve cost-effectiveness; however, further data protection requirements and software cost should also be accounted for.

### 3.6. Comparison with Previous Studies

Table 1 presents a summary of available studies regarding CVD screening in primary school children, including methodology, screening tools, diagnostic yield and cost-effectiveness when available.

## 4. Discussion

Early detection of CVD and related risk factors, already in childhood, is very important, as it allows early diagnosis, early treatment, preventive intervention and prevention of sudden cardiac death. The CVD screening program for primary school children in the Health Region of Crete represents one of the few structured programs targeting school-age children. This program is unique in terms of incorporating all available screening tools (including 12-lead ECG and digital phonocardiography) with data interpretation based on certified personnel [10] and use of designated EHR-CDDS for data entry and processing [17].

As CVD affecting children differs from adult CVD, characterized by a predominance of congenital heart disease (CHD) and rare forms of inherited cardiomyopathies/channelopathies in children [9] versus coronary artery disease (CAD) predominance in adults, CVD screening programs for children should be adapted accordingly, regarding both diagnostic targets and screening tools accordingly.

Undiagnosed CHD represents a rare (0.2%) cause of sudden cardiac death, with CHD affecting less than 1% of children [32]. Structural heart disease diagnosis is mainly based on the pediatrician’s clinical suspicion during routine evaluation/preparticipation screening or by the presence of symptoms (thoracic pain, easy fatigue, syncope etc.) [33,34]. The presence of a murmur in children represents a common referral indication for pediatric cardiology evaluation [35]. However, the majority corresponds to “innocent” murmurs [36,37,38], requiring no further evaluation other than expert auscultation [39]. However, the differentiation of innocent from abnormal murmurs and the detection of additional sounds associated with CHD can be challenging for non-experts, requiring clinical expertise or structured teaching [10].Telemedicine applications [15,38,40] or computer programs for automatic diagnosis and classification of cardiac sounds [19] can help clinical decision making. In the present screening program, digital phonocardiography (PCG) was applied based on our previous positive experience [10,15,17,18]. Following referral for abnormal auscultatory findings, a diagnosis of structural heart disease in 40 children followed. Nearly half of them corresponded to minor CHD forms (such as bicuspid aortic valve (BAV) and small atrial septal defect (ASD2) not requiring any intervention. The detection rate of structural heart abnormalities in the present CVD screening program was comparable to the results of screening programs using digital PCG in China [29], with variable detection rates depending on screening program methodology [24] (Table 1).

The detection of hereditary CVD including cardiomyopathies and channelopathies in childhood presents further challenges, due to their rarity and the limited diagnostic value of physical evaluation in this setting [41]. Accurate family history and the implementation and correct interpretation of pediatric 12-lead ECG are crucial for their early detection as they are associated with increased risk of SCD [42,43]. In our study, personal and family history information with potential association with the above diagnoses was documented, while 12-lead-ECG recording was also implemented. The detection of ECG abnormalities in 3% of screened children followed by further complex evaluation, correlates with the results of other schoolchildren screening programs in Europe [27,28,30,31] and in Asia [8,29] (Table 1). Significant ECG findings with potential association with increased SCD risk were documented in 0.2% of cases, with similar findings reported in an ECG screening program from Spain [27]. Despite the rarity of these diseases, ECG remains a first-line screening tool for identifying potentially lethal arrhythmogenic syndromes in asymptomatic patients.

An important goal of a CVD screening program in children is the early detection of risk factors associated with adult-onset CVD (such as CAD and hypertension). In the present study, the prevalence of hypertension and adiposity was 3% and 10%, respectively. The global prevalence of pediatric primary hypertension is increasing, affecting up to 4–5% of children [44], and being linked to major cardiovascular events in adulthood [45].Over the last half century, the estimated worldwide prevalence of obesity has increased to around 6% in boys and 8% in girls [46]. Childhood obesity (BMI > 95th percentile) and overweight (BMI > 85th < 94th percentile) are known independent CVD risk factors, linked to other risk factors such as elevated blood pressure [47]. Early detection and intervention of identifiable CVD risk factors in childhood represents a very important goal of any CVD screening program in childhood.

Finally, the present CVD screening program offered a unique chance of a general health evaluation of participant children as well, with the majority of referrals (13%) requiring further pediatric subspecialty care for their reported symptoms.

The cost-effectiveness of a CVD screening program in childhood should be viewed within the context of screening program goals (detection of risk factors, asymptomatic or symptomatic CVD), screening tools (history, physical evaluation, expert auscultation, 12-lead ECG) and available resources. The rarity of pediatric CVD alone cannot explain the limited availability of pediatric CVD screening programs (Table 1); inborn error of metabolism and other diseases which are targets of established screening programs (in the neonatal period) are more rare than the life-threatening forms of inherited and arrhythmogenic pediatric heart disease [9,48]. Based on the present study findings, CVD screening programs in childhood can be highly cost-effective in detecting CVD risk factors by using simple tools (weight, height, BP measurement) [49]. The diagnosis of minor CHD-structural abnormalities (incidence 1%) was based solely on expert cardiac auscultation, requiring personnel training and/or digital phonocardiography implementation costs. The diagnosis of electrocardiographic abnormalities (incidence 3%) was based only on 12-lead-ECG implementation, requiring further training for pediatric ECG interpretation or auto-interpretation algorithms [50]. Personal and family history responses are an integral part of preparticipation screening evaluation worldwide [12], and although easily obtained, they required expert confirmation while failing to document any diagnostic yield for CVD in the present study; the small sample size could account for this finding. However, positive history findings were very valuable for detecting children requiring further pediatric subspecialty evaluation as a cause of their symptoms, while positive responses of SCD in first-degree family members represented an indication for family cascade screening, including the family’s children.

The impact of seasonal and geographical variation on CVD and associated risk factors, as documented in the present study, deserves further study; clusters of rare inherited CVD such as cardiomyopathies and channelopathies can be detected, with affected regions deserving the highest inclusion priority in the design of pediatric CVD screening programs [9]. A potential seasonal impact, access to sports (reduced in rural areas) and temporal variations in physical activity (due to holidays or epidemic-related restrictions) could also account for the observed variability in CVD risk factors.

Future CVD screening programs in children could greatly benefit from the advantages of artificial intelligence, ECG and phonocardiogram auto-interpretation [19,51] and the implementation of electronic health records with decision-supported tools. Although we have extensively used digital phonocardiography and a designated EHR [17,19], the present study is still based on certified personnel participation for data sampling, confirmation and final diagnostic decision making [18].

### Study Limitations

The strengths of the present study include addresing a health issue (pediatric CVD screening) where only a small number of studies are available, providing valuable information for health care organizers to improve the effectiveness of similar screening programs and their implementation and a high potential for practical application. However, the present study findings should be viewed within the study limitations including limited statistical power due to small sample size to draw conclusions about rare CVDs such as channelopathies and the inclusion of a random sample of the reference population, with a subset of cases undergoing a final diagnostic evaluation. The study does not thoroughly compare the diagnostic yield of phonocardiography versus standard cardiac auscultation or 12-lead ECG. The applicability of the present study findings in other pediatric populations should also take into account geographical and ethnic differences in CVD prevalence [9], while further clarification of the performance of individual components of this screening program is needed. Finally, the study design does not include long-term outcomes of children diagnosed with risk factors or minor abnormalities further supporting the need for CVD screening programs in childhood.

## 5. Conclusions

CVD screening programs in school children can be very helpful for the early detection of CVD risk factors and their general health assessment as well. Expert cardiac auscultation and 12-lead ECG allow for the detection of structural and arrhythmogenic heard disease, respectively. Further study is needed regarding the performance of individual components, accuracy of interpretation (including computer-assisted diagnosis) and cost-effectiveness, before large-scale application of CVD screening in unselected pediatric populations.

## Figures and Tables

**Table 1:** CVD screening programs in primary school children.

Authors	Country	Duration	Methodology						Evaluated Children		Cost/ Effectiveness	Diagnostic Yield	
			Questionnaire	Auscultation	PCG	12-Lead ECG	BMI	BP	Number (N)	Age (Years)		ECG Abnormality (%)	Structural Heart Abnormality (%)
Tanaka et al., 2006 [20]	Kagoshima/Japan	1989–1997	yes	Yes	no	yes	yes	yes	37,807	12;15	**Yes**	0.005	0.013
Muta et al., 2003 [21]	Saga/Japan	1989–1998	yes	Yes	**yes**	yes	no	no	86,142 80,632	6;12	No data	No data	0.02 (ASD2 only)
Niwa et al., 2004 [22]	Chiba/Japan	1996–2001	yes	No	**no**	yes	no	no	71,855 80,467	6;12	No data	1.8	No data
Chiu et al., 2008 [23]	Taipei/Taiwan	1999–2001	yes	Yes	**yes**	yes	no	no	109,012 235,154	6;12	No data	0.84	No data
Yu et al., 2009 [24]	Taiwan	2005–2007	yes	yes	**yes**	yes	no	no	25,826	6;10; 13	No data	0.6	0.7
Yoshinaga et al., 2016 [25]	Kagoshima/Japan	2008–2013	yes	yes	no	yes	yes	yes	33,051 34,572	6;12	No data	6Y–LQTS 0.03 12Y–LQTS 0.09	No data
Liu et al., 2020 [26]	Taipei/Taiwan	2003–2014	yes	yes	**yes**	yes	no	no	566,478	6	No data	0.62	13.0
Dinarti et al., 2020 [8]	Indonesia	2015–2017	No	yes	no	yes	yes	yes	6116	9	No data	4.5	6.9
Vilardell et al., 2020 [27]	Girona/Spain	2009–2017	no	yes	no	yes	no	no	1911	13–14	No data	2.0	No data
Mancone et al., 2022 [28]	Italy	2017–2020	no	no	no	yes	no	no	11,949	13–19	No data	13.0	No data
Lim et al., 2023 [29]	Yunlin/China	2018–2019	Yes	no	**yes**	Digital ECG	no	no	1004	6–14	No data	2.2	4.5
Maurizi et al., 2023 [30]	Italy	2022	No	no	No	Digital ECG	no	no	728	12–13	**Yes**	3.0	No data
Greciano Calero et al., 2024 [31]	Balearic islands/Spain	2021–2022	yes	yes	no	yes	no	no	640	6;12	No data	3.1 at age 6Y 12.5 at age 12	No data
**Present Study**	Crete/Greece	2018–2023	**yes**	**yes**	**yes**	**yes**	**yes**	**yes**	944	8–9	**Yes**	3.0	4.2

## Data Availability

The data presented in this study are available from the corresponding author upon request. The data are not publicly available due to privacy and ethical restrictions.
